# CamKIIα and VPAC1 Expressions in the Caudal Trigeminal Nucleus of Rats After Systemic Nitroglycerin Treatment: Interaction with Anandamide

**DOI:** 10.3390/life15020155

**Published:** 2025-01-22

**Authors:** Gábor Nagy-Grócz, Eleonóra Spekker, Tamás Körtési, Klaudia Flóra Laborc, Zsuzsanna Bohár, Annamária Fejes-Szabó, László Vécsei, Árpád Párdutz

**Affiliations:** 1 Department of Theoretical Health Sciences and Health Management, Faculty of Health Sciences and Social Studies, University of Szeged, Temesvári Krt. 31, H-6726 Szeged, Hungary; nagy-grocz.gabor@szte.hu (G.N.-G.); kortesi.tamas@szte.hu (T.K.); 2 Preventive Health Sciences Research Group, Incubation Competence Centre of the Centre of Excellence for Interdisciplinary Research, Development and Innovation of the University of Szeged, H-6720 Szeged, Hungary; 3 Competence Centre for Drug Development and Clinical Trials, Centre of Excellence for Interdisciplinary Research, Development and Innovation, Korányi Fasor 6, H-6720 Szeged, Hungary; spekker.eleonora@gmail.com; 4 HUN-REN-SZTE Neuroscience Research Group, University of Szeged, Semmelweis u. 6, H-6725 Szeged, Hungary; zsuzsanna.bohar@gmail.com (Z.B.); fejesannamaria@yahoo.co.uk (A.F.-S.); 5 Neuropathology Brain Bank & Research CoRE, Department of Pathology, Molecular and Cell-Based Medicine, Icahn School of Medicine at Mount Sinai, New York, NY 10029, USA; claudia.laborc@mountsinai.org; 6 Department of Neurology, Albert Szent-Györgyi Medical School, University of Szeged, Semmelweis u. 6, H-6725 Szeged, Hungary; apardutz@yahoo.com

**Keywords:** migraine, trigeminal system, nitroglycerin, anandamide, CamKIIα, PACAP, VIP

## Abstract

Migraines are a frequently occurring neurological condition that affects up to 16% of the global population. The precise pathomechanism of the disease remains unknown, but from animal and human observations, it appears that calcium/calmodulin-dependent protein kinase II alpha (CamKIIα), pituitary adenylate cyclase-activating polypeptide (PACAP), and vasoactive intestinal polypeptide (VIP) are involved in its pathogenesis. One of the animal models of migraines uses the systemic administration of nitroglycerin (NTG), which, as a nitric oxide (NO) donor, initiates a self-amplifying process in the trigeminal system, leading to central sensitization. Endocannabinoids, such as anandamide (AEA), are thought to play a modulatory role in trigeminal activation and sensitization phenomena. In the present experiment, we aimed to investigate the effect of NTG and AEA on CamKIIα, PACAP/VIP, and vasoactive intestinal polypeptide type 1 receptor (VPAC1) expression levels in the upper cervical spinal cord (C1-C2) of rats, where trigeminal nociceptive afferents are clustered. Four groups of animals were formed: in the first group, the rats received only the vehicle; in the second group, they were treated with an intraperitoneal injection of NTG (10 mg/kg); animals in the third and fourth groups received AEA (2 × 5 mg/kg) half an hour before and one hour after the placebo or treatment with NTG. Four hours after the placebo/NTG injection, the animals were transcardially perfused, and the cervical spinal cords were removed for Western blot. Our results show that both NTG and AEA alone can increase the expression of CamKIIα and VPAC1 in the C1-C2 segments. Interestingly, the combination of NTG and AEA had no such effect on these markers, possibly due to various negative feedback mechanisms.

## 1. Introduction

Migraines are a chronic neurological disorder affecting up to 16% of the global population, characterized by recurrent headaches lasting from 4 to 72 h that are commonly accompanied by nausea, photophobia, and phonophobia [1].

The fact that systemic nitroglycerin (NTG) is able to generate migraine attacks in migraineurs [2] and activates and sensitizes the trigeminal system both in humans [3] and animals [4] makes it an ideal model for the disease. Besides the morphological changes, NTG can cause behavioral changes within 3–6 h in the rat, strengthening its use as a migraine model. It is known that NTG reduces the locomotor activity of the rats in open field tests [5] and induces mechanical thermal hyperalgesia, place aversion, and photophobia [6,7], which are comparable to the symptoms of human migraine.

Calcium/calmodulin-dependent protein kinase II alpha (CamKII) plays an important role in the nociceptive processing and the sensitization of central sensory neurons [8], which is essential in the development of the attacks [9]; thus, CamKII might contribute to migraine pain [10]. It is also known that central sensitization of the spinal cord neurons depends on the levels of CamKIIα [11], which can be persistently activated by autophosphorylation [12].

Pituitary adenylate cyclase-activating polypeptide (PACAP) is a member of the vasoactive intestinal polypeptide/secretin/glucagon peptide family [13], and it is present in the superficial layers of the cervical spinal cord [14]. Similarly to NTG, it can provoke a dull headache in healthy people and generate migraine attacks in migraineurs [15]. In addition to this, PACAP and its receptors have a role in the activation and sensitization process of the trigeminal system both in animals and in humans. PACAP-38 immunoreactivity levels were significantly lower in the interictal plasma of migraineurs compared to healthy controls but higher during migraine attacks [16]. In rats, the levels of both PACAP-38 and PACAP-27 immunoreactivity increase selectively in the caudal trigeminal nucleus in response to chemical and electrical stimulation of the trigeminal afferents [17].

PACAP acts via G-protein–coupled receptors, such as vasoactive intestinal polypeptide receptor type 1 and 2 (VPAC1, VPAC2) and pituitary adenylate cyclase–activating polypeptide receptor type 1 (PAC1). A previous study found that PAC1 expression does not change after the administration of NTG [17]. Thus, we concentrated on the other PACAP receptor: VPAC1.

*Cannabis* is well known for its antiemetic and antinociceptive properties; it has been employed for a long time to reduce chemotherapy-induced nausea and vomiting and to treat pain, migraine, and muscle spasticity [18]. Anandamide (AEA) is an extensively studied endocannabinoid that effectively inhibits trigeminal activation and central sensitization in animals [19,20,21]. Cannabinoid receptors (mainly CB1 and CB2) are expressed in areas associated with pain modulation, including the trigeminal system [22]. The activation of these receptors by endocannabinoids or exogenous cannabinoids (e.g., THC) can inhibit the release of pro-inflammatory neuropeptides, such as calcitonin gene-related peptide (CGRP) [23], which is heavily involved in migraine pathogenesis [24].

The aim of the present study was to investigate the effects of NTG and AEA on CamKIIα and VPAC1 expression levels in the superficial lamina of the upper cervical spinal cords (C1-C2) in rats using Western blot.

## 2. Materials and Methods

### 2.1. Animals

The procedures utilized in this study followed the guidelines for the Use of Animals in Research of the International Association for the Study of Pain and the directive of the European Economic Community (86/609/ECC). They were approved by the Animal Research Committee of the University of Szeged and the Scientific Ethics Committee for Animal Research of the Protection of Animals Advisory Board (XXIV./352/2012, 14 June 2013). Twenty adult male Sprague–Dawley rats weighing 200–250 g were used. The animals were raised and maintained under standard laboratory conditions, with tap water and regular rat chow available ad libitum in a 12 h dark/12 h light cycle.

### 2.2. Drug Administration

The animals were randomly divided into four groups (*n* = 5 per group). The rats in the first group (placebo) received only the vehicle solutions as treatment. In the second group, the rats were treated with an intraperitoneal injection of NTG (10 mg/kg body weight, Pohl Boskamp, Hohenlockstedt, Germany). In the third and fourth groups, the rats were given AEA (2 × 5 mg/kg) injection half an hour before and one hour after the placebo or NTG treatment.

### 2.3. Western Blotting

Four hours after the placebo/NTG injection, the animals were transcardially perfused with 100 mL PBS; then, the dorsal horns of the C1-C2 segments were extracted. The samples were stored at −80 °C until use. Before measurements were taken, the specimens were sonicated in an ice-cold lysis buffer containing 50 mM Tris-HCl, 150 mM NaCl, 0.1% igepal, 0.1% cholic acid, 2 μg/mL leupeptin, 2 mM phenylmethylsulphonyl fluoride, 1 μg/mL pepstatin, 2 mM EDTA, and 0.1% sodium dodecyl sulfate. The lysates were centrifuged at 12,000 rpm for 10 min at 4 °C, and the supernatants were aliquoted and stored at −20 °C until use. Protein concentration was defined with the BCA Protein Assay kit (Thermo Fischer Scientific, Waltham, MA, USA) using bovine serum albumin as a standard. Prior to loading, each sample was mixed with sample buffer and denatured by boiling for 3 min. The protein content of the samples was measured, and equal volumes of protein sample (20 μg/lane) were separated by standard SDS-PAGE on 10% Tris–glycine gel and electrotransferred onto an Amersham Hybond-ECL nitrocellulose membrane (0.45 μm pore size, GE Healthcare, Chicago, IL, USA). Gel electrophoresis was performed in parallel with the analyzed marker and GAPDH. We used a 10–180 kDa PageRuler Prestained Protein Ladder (Thermo Fischer Scientific, Waltham, MA, USA) to determine estimated molecular weights. Following the transfer, membranes were blocked for one hour at 20 °C in Tris-buffered saline containing Tween 20 (TBST) and 5% non-fat dry milk. The membranes were then incubated in TBST containing 1% non-fat dry milk and CamKIIα (Sigma-Aldrich, St. Louis, MO, USA, C-265, dilution: 1:4000, incubation: overnight at 4 °C) or VPAC1 antibody (Santa Cruz Biotechnology, Dallas, TX, USA, sc-52794, dilution: 1:1000, incubation: overnight at 20 °C) or glyceraldehyde 3-phosphate dehydrogenase (GAPDH) antibody (Thermo-Fischer Scientific, Waltham, MA, USA, 26617, dilution: 1:1000, incubation: overnight at 20 °C). The following day, the membranes were incubated in TBST containing 1% non-fat dry milk and horseradish peroxidase-conjugated anti-rabbit or anti-mouse secondary antibodies (Santa Cruz Biotechnology, Dallas, TX, USA, sc-2030, sc-2031) for two hours at 20 °C. Protein bands were visualized after the incubation of the membranes with SuperSignal West Pico Chemiluminescent Substrate using Carestream Kodak BioMax Light film.

### 2.4. Data Evaluation

A blinded observer assessed all data. Films were scanned and quantified for densitometric analyses using Java ImageJ 1.47v analysis software (National Institute of Health, Bethesda, MD, USA). GAPDH served as a control to ensure the loading of equivalent amounts of sample proteins, and the results were normalized to GAPDH.

### 2.5. Statistical Analysis

Statistical analysis was performed via SPSS Statistics software (Version 20.0 for Windows, SPSS Inc., Chicago, IL, USA) using one-way analysis of variance followed by Fisher’s least significant difference, with *p* < 0.05 taken as statistically significant. Group values are reported as means ± SEM.

## 3. Results

### 3.1. NTG and AEA Enhance the Expression of CamKIIα in the C1-C2, Which Is Not Present After NTG + AEA Treatment

**Figure 1:**
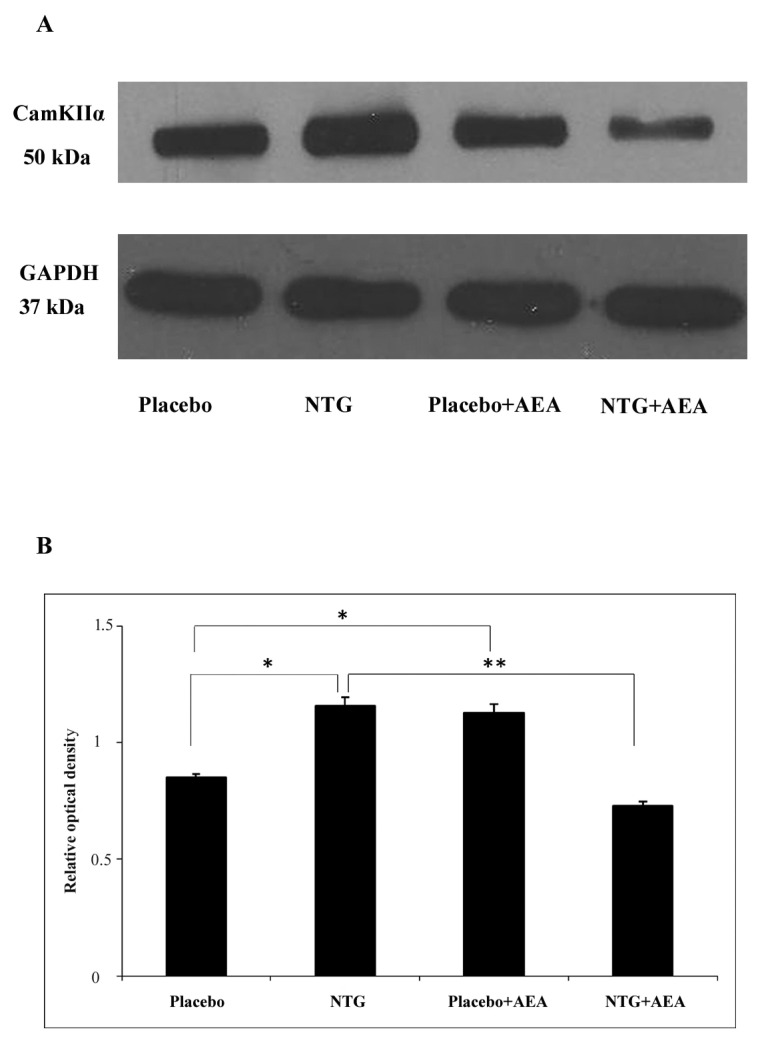
Effect of NTG and AEA on the expression of CamKIIα, Western blot data. (**A**). Western blotting of CamKIIα and GAPDH expression in the C1-C2. (**B**). Densitometry of the individual bands showed that in NTG- and AEA-treated animals, the expression of CamKIIα was significantly higher compared to the placebo group. No such effect was observed in the NTG + AEA-injected animals. * *p* < 0.05; ** *p* < 0.01.

A band characteristic of the CamKIIα protein was detected at 50 kDa in the Western blot assay. Densitometric analyses confirmed that the CamKIIα bands were significantly enhanced (*p* < 0.05) in segments C1-C2 after NTG and AEA administration compared with placebo-treated animals. This effect was not seen after the combined treatment with NTG + AEA (*p* < 0.01) (Figure 1).

### 3.2. NTG and AEA Enhance the VPAC1 Expression in the C1-C2, but Combined NTG + AEA Treatment Did Not

**Figure 2:**
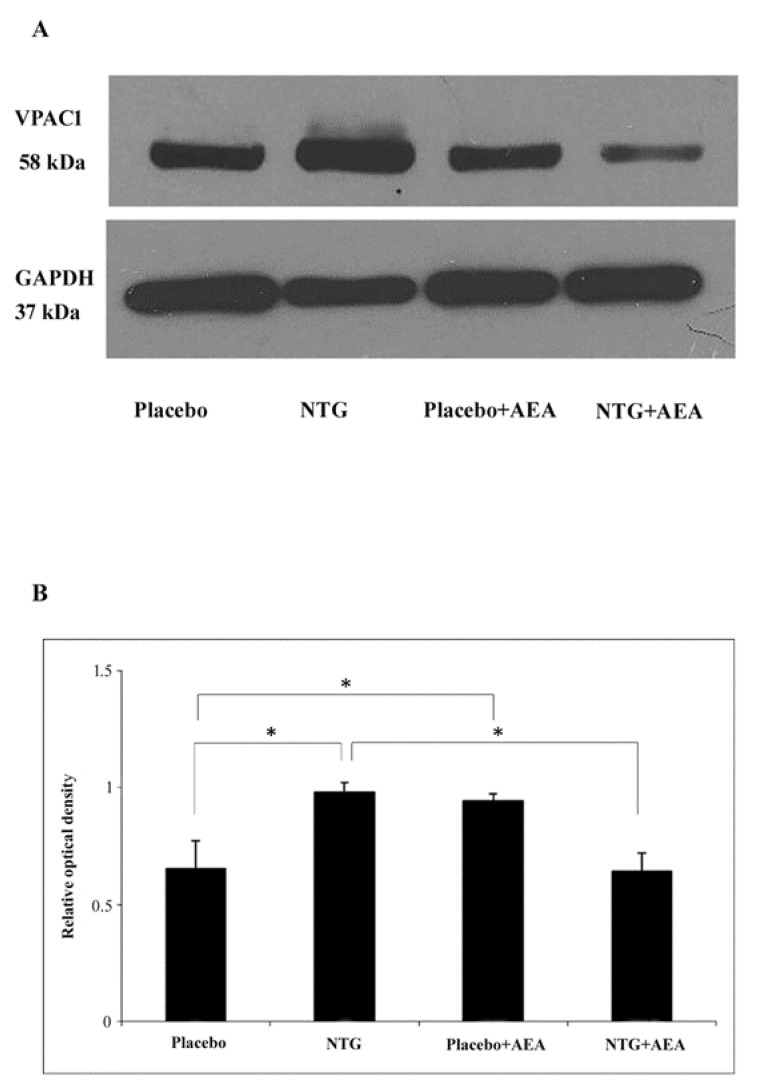
Effect of NTG and AEA on the expression of VPAC1, Western blot data. (**A**). Western blotting of VPAC1 and GAPDH expression in the C1-C2. (**B**). Quantitative data demonstrate, that the relative optical density of VPAC1 in the NTG and AEA groups is significantly higher than in the placebo group. NTG + AEA mitigated this effect. * *p* < 0.05.

A band characteristic of the VPAC1 protein was identified at 58 kDa in the Western blot assay. Densitometric analyses showed that the VPAC1 bands were significantly enhanced (*p* < 0.05) in segments C1-C2 after NTG and AEA administration compared to the placebo-treated animals. This effect was decreased by the combined NTG + AEA treatment (*p* < 0.05) (Figure 2).

## 4. Discussion

Trigeminal activation and sensitization are essential phenomena associated with migraine attacks [25], where both NO and AEA play an important role. NO may activate the trigeminovascular system [26], leading to sensitization. Endocannabinoids, such as AEA, affect this activation mainly by interacting with neuroinflammatory mediators, such as CGRP, which mRNA expression is upregulated after AEA [27]. On the other hand, it has also been revealed that AEA can inhibit the responses of trigeminal neurons in rats [28], strengthening the possible role in the inhibition of the trigeminal system. It is important to mention that synaptic transmission is often regulated by the activation of protein kinases, such as cyclic AMP (adenosine monophosphate)-dependent protein kinase A (PKA). Activation of CB1 receptors (G protein-coupled receptors) by AEA leads to inhibition of adenyl cyclase [29], the enzyme responsible for converting adenosine triphosphate into cAMP. By inhibiting adenylyl cyclase, AEA reduces intracellular cAMP levels [30], resulting in a downstream decrease in the activation of PKA and its related signaling pathway. PKA can influence synaptic activity by modifying postsynaptic properties, but it also plays a role in controlling presynaptic functions [31,32]. When PKA is inhibited, it decreases the release of neurotransmitters from many presynaptic terminals. PKA targets include presynaptic ion channels, and its activation may influence calcium levels within the presynaptic terminals [33].

In the present study, we investigated the effect of NTG and AEA administration on the expression of CamKIIα and VPAC1, markers of importance in the activation and sensitization of the trigeminal system, thus in the pathogenesis of migraine.

Similarly to previous observations [34] in our present experiment, NTG increased CamKIIα expression in the C1-C2 segments, receiving the majority of nociceptive input from the trigeminal system. This phenomenon is parallel to the changes in neuronal nitric oxide synthase (nNOS) increase in the same area related to the activation of small-caliber sensory fibers by NO, leading to a self-amplifying process, reflecting a central sensitization process there [35]. Moreover, CamKIIα expression in the superficial laminae of the C1-C2 is increased after subcutaneous formalin injection [8] and capsaicin administration [11], which can also be considered as an activation/sensitization trigger in the trigeminal system. Furthermore, it has been shown that nociceptive stimuli (hind paw capsaicin injection) upregulate CamKIIα expression in the dorsal horns of rats [36]. These data obviously show that CamKIIα (and its phosphorylation) is one of the key elements in the sensitization process of the nociceptive pathway.

The results of the present experiment demonstrate that NTG increased VPAC1 expression in the C1-C2 segments, as well. VPAC1 is one of the receptors of PACAP and is located in the liver [37], lung [37], and CNS [38]. PACAP has two biologically active forms: PACAP-27 and PACAP-38 [39]; the latter increases the diameter of the superficial temporal arteries [40]. Both functional isoforms generate migraine attacks in migraineurs [40,41]. NO donor NTG failed to change the meningeal blood flow and to increase the c-Fos expression in PACAP knock-out mice compared to the wildtype mice, underlining the role of PACAP in the pathology of migraine and its importance in central and peripheral sensitization processes in these animals [17].

Clinical studies suggest that PACAP and its receptors play a key role in primary headache disorders, making PACAP signaling a potential target for migraine treatment [42]. PACAP infusion caused headaches and vasodilation in both healthy individuals and migraine patients and increased levels of relevant disease markers [40,43]. However, a phase 2 study using AMG 301, a monoclonal antibody targeting the PAC1 receptor, showed no significant effect on migraine prevention [44]. In contrast, another PACAP ligand antibody, Lu AG09222, significantly reduced migraine days [45]. While more research is needed, PACAP might be promising as a possible future migraine treatment.

It is widely recognized that the VPAC1 receptor induces vasodilation by producing nitric oxide (NO) [46], which enhances the activation of cyclic guanosine monophosphate (cGMP) [47] and cAMP pathways [48].

Subsequently, cGMP and cAMP activate PKA and PKC, which have been shown to phosphorylate eNOS [49]. However, there is some cross-talk between the cGMP and cAMP pathways, meaning that under certain conditions, the increase in cGMP can indirectly influence cAMP levels or its effects. cGMP modulates the concentration of cAMP by activating or inhibiting cAMP-specific phosphodiesterases (PDEs) [50]. PACAP can also induce the activation of cAMP [51]. Although PACAP primarily increases cAMP levels, there is potential cross-talk between the cAMP and cGMP pathways. This means that an increase in cAMP (due to PACAP) could indirectly influence cGMP signaling, as PDEs that break down both cAMP and cGMP might mediate this interaction [52]. In summary, we speculate that there is cross-talk between PACAP and NO, resulting in a positive feedback mechanism that may indicate central sensitization. On the other hand, NO can increase the release of CGRP by influencing voltage-gated calcium channels [53] and increasing cGMP levels [54] that regulate neurotransmitter exocytosis. Furthermore, NO can modulate transient receptor potential vanilloid 1 (TRPV1) receptors through nitrosylation [55], the covalent attachment of a NO group to cysteine residues in proteins. This modulation can contribute to the sensitivity of TRPV1 receptors under pathological conditions, such as inflammation [56], where NO is produced in higher amounts.

We found that AEA alone can also increase the expression of CamKIIα and VPAC1. It is well-known that AEA activates TRPV1 receptors, causing an increased intracellular calcium level and initiating CamKII signaling [57]. Meanwhile, AEA can also induce Ca^2+^ increase via G-proteins and thus enhance CamKIIα expression in the C1-C2 segments [58,59].

The VPAC1 receptor has a similar affinity to PACAP and VIP, which release is related to an NO- and AEA-dependent mechanism [60]. VIP liberation is increased in migraine patients during attacks [61]; moreover, NO release is functionally coupled to VIP secretion under certain conditions [62], and it can increase the levels of NO, too [63]. Thus, we may assume that VIP can also modulate VPAC1 since it can exert its effect on both VPAC1 and VPAC2 receptors [64]. In addition to this, AEA can also increase the levels of VIP in N18 neuroblastoma cells [65] and nerve terminals in rat ileum [60]. The increased VIP levels can also influence VPAC1 by binding to its receptor and increasing cAMP concentrations [66]. To summarize these data, we can conclude that both NO and AEA can influence the VPAC1 receptors through numerous pathways (NO, AEA, PACAP, and VIP), which can explain the complex interactions between these systems.

On the other hand, we cannot exclude a more direct link between CamKII and VPAC1 either. PACAP promotes glutamatergic transmission in the amygdala through VPAC1 receptors, which can be inhibited by autocamtide-2-related inhibitory peptide [67], a selective and potent CamKII inhibitor, suggesting a possible direct connection between CamKII and VPAC1. CamKII is also important in the cellular synchronization among oscillators in the suprachiasmatic nucleus (SCN) [68], which is responsible for the daily behavioral rhythms or central circadian clock in mammals and requires VPAC receptors for a normal function [69] also reflecting a strong interaction between the two systems.

**Figure 3:**
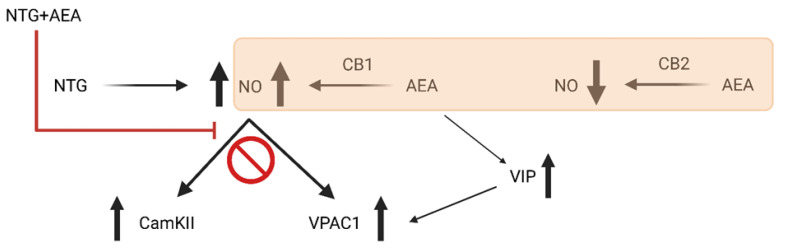
Possible mechanisms of NTG and AEA on the CamKIIα and VPAC1 expression. Both NTG and AEA can increase the expression of CamKIIα and VPAC1, probably by generating NO production. NO can initiate the phosphorylation of CamKIIα and can activate adenylate cyclase, which also triggers the production of PACAP. In addition to this, AEA also promotes the formation of NO by the CB1 receptor. On the other hand, the combined treatment might cause a huge boost in NO production, which activates a negative feedback mechanism and decreases the levels of NO in the long run. Also, it is well known that AEA can also reduce the synthesis of NO via the CB2 receptor.

It is puzzling that the combined treatment in our experiment is not able to modify the expression of the examined markers in the upper cervical spinal cord of rats. We hypothesized the following possibilities: The first one is that NTG and AEA alone are able to increase the release of NO (and thus activate nNOS), and the combined treatment can boost a negative feedback mechanism that attenuates the changes. A similar effect has been found in rat enteric synaptosomes and in gastric myenteric plexus, where exogenous NO can inhibit its endogenous production in a dose-dependent manner [70,71]. On the other hand, it is also possible that the combined treatment (high levels of NO) can desensitize TRPV1 receptors [72]. Thus, AEA inhibits TRPV1-related influx of Ca^2+^, decreasing the expression of CamKIIα. This hypothesis is strengthened by Lau and co-workers, since they found that the desensitization of TRPV1 depends on the calcium-calmodulin interactions [73]. Besides that, it should be noted that AEA can activate NOS enzymes (production of NO) through cannabinoid receptor type 1 (CB1), while AEA is also able to inhibit NOS enzymes via CB2 [74,75]. Thus, we cannot exclude the fact that this dual effect contributes to our results. In addition to this, several research data point out that NO can influence CB receptors. The NO donor S-nitroglutathione can inhibit the CB1 receptor in rat cerebral cortex and hippocampus, probably by NO-generated (S-nitroglutathione) post-translational modification of CB1 [76]. In the other experiment, NO influenced CB2 receptor gene transcription during neuropathic pain in rats [77], suggesting a direct action of NO on this molecule. Taken together, we can conclude that this is a connection between NO and CB receptors, and this link probably also has an influence on our results (Figure 3).

Summarizing the results, it can be stated that NTG was able to influence the expression of CamKII alpha and VPAC1, which are essential during a migraine attack. Furthermore, AEA was able to inhibit these effects. Interestingly, AEA also had an impact on the studied markers on its own. Since we have indirect evidence, a limitation of the study is that the exact mechanism of AEA effects remains not fully known. Based on the available data, it is likely that AEA exerted the observed effects either through the activation of cannabinoid receptors or via a negative feedback mechanism. Further experiments are needed to clarify this question.

## 5. Conclusions

The results of the present study may help to (1) explore the connection between the endocannabinoid system and the NO system and (2) examine the trigeminal activation and sensitization process. An important question that can be raised is how combined treatment could decrease the expression of the examined markers. We hypothesized that it is associated with a negative feedback mechanism, but it is also possible that the observed changes are due to the “Janus face” of AEA since it has a dual action on the NO system. This makes AEA a potential candidate for migraine therapy, as enhancing its effects could counteract the migraine-inducing actions of NO and alleviate symptoms such as headache and inflammation. The limitation of this study is that we cannot fully explain the observed changes. Further investigations will be required in the future, such as examining cannabinoid receptor inhibitors or assessing the effects of chronic NTG treatment on these markers.

## Data Availability

The original contributions presented in this study are included in the article. Further inquiries can be directed to the corresponding author.
